# Model-Aided Localization and Navigation for Underwater Gliders Using Single-Beacon Travel-Time Differences

**DOI:** 10.3390/s20030893

**Published:** 2020-02-07

**Authors:** Jie Sun, Feng Hu, Wenming Jin, Jin Wang, Xu Wang, Yeteng Luo, Jiancheng Yu, Aiqun Zhang

**Affiliations:** 1 State Key Laboratory of Robotics, Shenyang Institute of Automation, Chinese Academy of Sciences, Shenyang 110016, China; sunjie@sia.cn (J.S.); hufeng@sia.cn (F.H.); jinwm@sia.cn (W.J.); wangjin@sia.cn (J.W.); wangxu1@sia.cn (X.W.); luoyeteng@sia.cn (Y.L.); zaq@sia.cn (A.Z.); 2 Institutes for Robotics and Intelligent Manufacturing, Chinese Academy of Sciences, Shenyang 110169, China; 3 University of Chinese Academy of Sciences, Beijing 100049, China

**Keywords:** underwater gliders, modified kinematic model, travel-time difference, EKF estimation, RTS smoothing

## Abstract

An accurate motion model and reliable measurements are required for autonomous underwater vehicle localization and navigation in underwater environments. However, without a propeller, underwater gliders have limited maneuverability and carrying capacity, which brings difficulties for modeling and measuring. In this paper, an extended Kalman filter (EKF)-based method, combining a modified kinematic model of underwater gliders with the travel-time differences between signals received from a single beacon, is proposed for estimating the glider positions in a predict-update cycle. First, to accurately establish a motion model for underwater gliders moving in the ocean, we introduce two modification parameters, the attack and drift angles, into a kinematic model of underwater gliders, along with depth-averaged current velocities. The attack and drift angles are calculated based on the coefficients of hydrodynamic forces and the sensor-measured angle variation over time. Then, instead of satisfying synchronization requirements, the travel-time differences between signals received from a single beacon, multiplied by the sound speed, are taken as the measurements. To further reduce the EKF estimation error, the Rauch-Tung-Striebel (RTS) smoothing method is merged into the EKF system. The proposed method is tested in a virtual spatiotemporal environment from an ocean model. The experimental results show that the performance of the RTS-EKF estimate is improved when compared with the motion model estimate, especially by 46% at the inflection point, at least in the particular study developed in this article.

## 1. Introduction

Underwater gliders are a type of autonomous underwater vehicles (AUVs) but without a propeller [1], which are great autonomous platforms fitted in a persistent underwater surveillance system [2,3]. Due to the limited carrying capacity restricted by the design of underwater gliders, unless specifically required, underwater gliders are not equipped with sensors such as an inertial measurement unit (IMU) [4], a Doppler velocity log (DVL) or an acoustic Doppler current profiler (ADCP) sonar, which are usually carried by AUVs [5]. This makes the navigation and positioning of underwater gliders more challenging than for AUVs, but obtaining the glider’s position is very critical for tasks such as target detection [6] and sound field construction [7].

A collection of localization and navigation methods have been proposed for AUVs [8,9,10,11,12]. Any navigation algorithm is based on state estimation [8], which is conducted in a predict-update cycle. The prediction process of state estimation is established mainly based on motion models of AUVs. Based on the installed sensors, AUVs usually adopt inertial navigation by using the IMU-detected acceleration and the DVL-measured relative velocity of the AUV [13]. A carried ADCP provides the relative speed of the local current for AUV navigation [14]. The navigation of an underwater glider depends on the glider’s working mechanism and the installed sensors, including electronic compass and conductivity-temperature-depth (CTD) sensors, but without any velocity sensor. Based on the working mechanism, the dynamic motion of underwater gliders can be modeled [15,16,17]. For practical applications, motion models are usually simplified, for example, applying Lanchester’s phugoid assumptions [18,19] which assuming the angle of attack is fixed [20]. In [21], a localization scheme is proposed for underwater gliders by neglecting the influence of ocean currents and by assuming a zero drift angle. Seaglider and the Sea-Wing underwater glider have the ability to estimate the depth-averaged current velocities for engineering applications [22,23], which could be utilized for a glider localization and navigation scheme. Here, we combine a kinematic model of underwater gliders with the estimated attack and drift angles and add the depth-averaged current velocities [24] to model the prediction process of the glider positions in the ocean.

Measurements are needed for the updating process to correct the prediction errors. For AUVs, in addition to relying on the installed angle and velocity sensors, the acquisition of measurements depends mainly on acoustic beacons and geophysical features [9]. Geophysical techniques use external environmental information as a reference, which can be observed by sonar [25,26,27], optical [28] or magnetic [29] sensors fitted to AUVs. Acoustic techniques are focused on different types of acoustic features, such as the travel time [30,31] and time difference of arrival [32,33], resulting from hydrophone signals. Common measuring techniques include the ultrashort baseline (USBL), short baseline (SBL), long baseline (LBL), single-beacon and acoustic modem techniques [8]. For USBL and SBL navigation, a set of transceivers and transponders is required, one mounted on the AUV and the other mounted on the support vehicle [34,35]. For LBL or single-beacon navigation, one-way travel-time ranging can be conducted on the basis of synchronized clocks [36,37]. For acoustic communication-based underwater navigation, an acoustic communication system is needed for both the transmitter and the AUV receiver [38]. To reduce the requirements for sensors on underwater gliders, a single beacon is selected as the measuring technique. Regarding the glider’s movement over time as a virtual array, we adopt the travel-time difference between signals from the beacon received at two adjacent receiving locations as the measuring acoustic feature, which conventionally refers to the time difference of arrivals at a pair of hydrophones in an acoustic array [39,40]. Herein, it is assumed that the acoustic beacon transmits signals at a regular interval of time instead of satisfying synchronization requirements and that the beacon remains stationary.

Various methods have been used to solve the predict-update cycling state estimation, mainly including the least squares [41], extended Kalman filter (EKF) [42,43], unscented Kalman filter [44], extended information filter [45], and particle filter [46,47] methods. Considering that our proposed algorithm can be performed online, we select an EKF method for the state estimation of underwater gliders, which can approximate nonlinear systems by a linearized first-order Taylor series expansion [48]. The EKF is employed to fuse acoustic measurements and predictions of the process model [49]. Specifically, estimating the glider adjacent positions from the process model enables the algorithm to properly model the range-difference measurements, and the glider position predicted by the process model is corrected within the EKF framework by real range-difference measurements.

Smoothing, which can be viewed as the third part for sequential estimation of glider’s positions, differs from filtering in that further measurements are assimilated [50,51]. Therefore a smoothing process intuitively reduces the EKF estimation error. Many forms of smoothing algorithms have been developed, in which the two-filter smoother and the forward-backward smoother are usually used when numerical approximations are required [52]. Forward-backward smoothers process the measurements first by a forward filter to compute the filtered estimate, and then by a backward smoothing pass to determine the smoothing estimate from the forward filtered estimate [53]. The two-filter smoothing is an alternative approach to the forward-backward smoothing, relying on two independent filter procedures: one runs forward in time and another runs backward [54]. However, the backward filter for the two-filter smoother usually cannot be computed in a closed form, which prohibits the use of numerical techniques to approximate the smoothing [55]. Hence we adopt a forward-backward smoothing method, named the Rauch-Tung-Striebel (RTS) smoothing method [56], for smoothing estimation. The RTS smoother has been widely employed in inertial navigation systems [57] and target tracking problems [58,59].

Our main innovations are as follows. First, we establish a modified kinematic model of underwater gliders by introducing the attack and drift angles estimated by hydrodynamic coefficients and angles measured by electronic compass or derived from control system records. Second, the range variation from underwater gliders to a static acoustic beacon, calculated from the travel-time difference of two adjacent signals from the beacon, is set as the measurement for the EKF-based localization and navigation system.

The rest of the paper is organized as follows. We describe the localization and navigation issues of underwater gliders in Section 2. In Section 3, we theoretically derive the attack and drift angles to modify a kinematic model of underwater gliders. In Section 4, an EKF-based localization and navigation system is constructed by introducing the depth-averaged currents to the modified kinematic model and calculating the discrete ranging differences as the measurements. The RTS smoothing method is used to further improve the EKF estimate. A simulation experiment is conducted in Section 5. The state data of underwater gliders are based on the records recorded during an experiment conducted in the South China Sea, including angles measured by electronic compass, depth information measured by CTD, and rudder angles derived from control system records. Spatiotemporal ocean currents are generated from an ocean model. The travel times of acoustic signals are simulated via an acoustic simulator based on a spatiotemporal environmental field from an ocean model. In Section 6, we present discussion and conclusions.

## 2. Problem Statement

**Figure 1:**
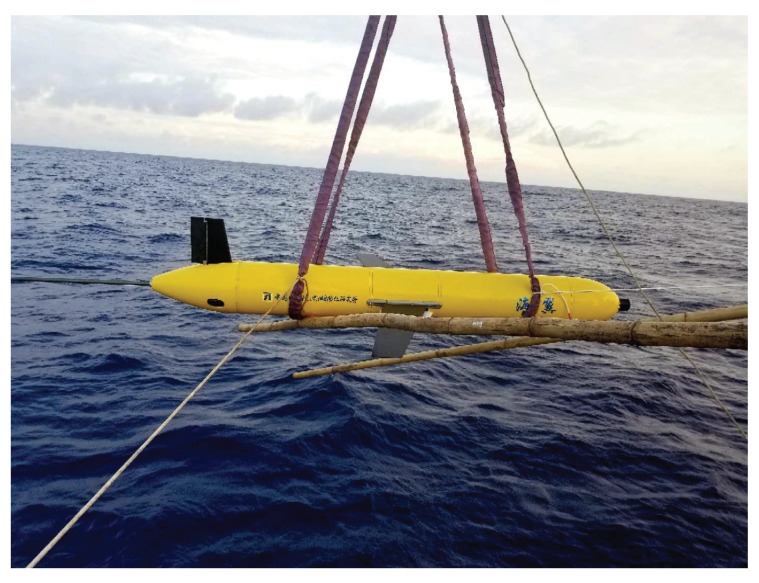
The acoustic Sea-Wing underwater glider.

The Sea-Wing underwater glider, developed by the Shenyang Institute of Automation, Chinese Academy of Sciences, is shown in Figure 1, and some of the specifications of the Sea-Wing underwater glider are summarized in Table 1. The processes related to posture adjustment on the Sea-Wing underwater glider include buoyancy, pitch and rudder regulations [60]. The gliders move vertically by adjusting their buoyancy and generate a gliding motion through the ocean via a pair of wings. The driving buoyancy is controlled by altering the volume of the underwater glider by feeding hydraulic oil into or bleeding hydraulic oil out of an external oil bladder. The pitch, which determines the vertical gliding angle, is controlled by moving an internal battery package along the longitudinal axis to change the center of gravity of the glider. The rudder, installed on the tail of the glider, alters the course angle by changing the rudder angle to resist the effects of ocean currents. Therefore, to describe the movement of underwater gliders, we need to involve buoyancy, pitch and rudder regulating processes.

The sensors mounted on the Sea-Wing underwater glider provide an effective tool for measuring the status of the glider. The CTD sensor and electronic compass are the two most important sensors for glider navigation in the ocean environment. Through the depth change of the glider over time as measured by the CTD, the vertical speed of the glider in the inertial coordinate system can be estimated, which reflects the buoyancy regulation. From the measurements of electronic compass, the pitch and rudder regulating processes can be incorporated into the glider motion simulation. Furthermore, to simulate the glider motion in the real ocean environment, the external forces and force variations for the glider also need to be considered. The hydrodynamic model is an excellent tool to simulate and analyze the forces of underwater gliders. Based on the glider-mounted sensors and hydrodynamic parameters, we can simulate the glider motion by an improved kinematic model.

**Figure 2:**
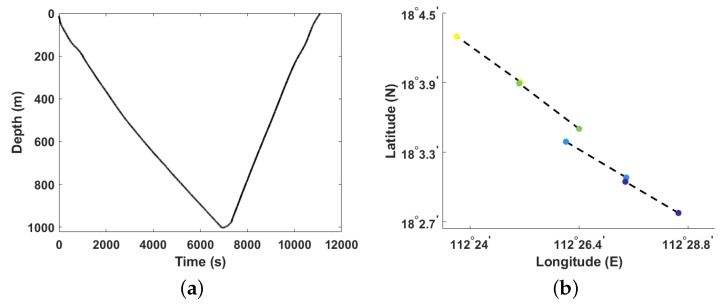
Examples of a glider’s trajectories in the South China Sea on 13 July 2019. (**a**) The glider depth change in the vertical plane with the relative time in a gliding cycle. (**b**) Circles with the same color represent global positioning system (GPS) positions of the glider at the sea surface at the beginning and end of a gliding cycle, circles with different colors represent different cycles, and dashed lines represent the linearly interpolated horizontal positions during a gliding cycle.

A geodetic global positioning system (GPS) receiver is installed on the underwater glider for implementing glider positioning at the sea surface. However, the gliders cannot rely on sensors on board for positioning while underwater. Figure 2 shows an example of the glider’s trajectories. On a vertical section, we can only acquire the glider’s depth over time, instead of over latitude and longitude coordinates. On a horizontal section, we can localize the glider at the sea surface only.

Based on the proposed kinematic model, we can estimate the position of underwater gliders in still water, whereas the flow influence in the ocean is inevitable. With the depth-averaged current velocity estimation module on the glider, the positions of underwater gliders in the ocean can be roughly estimated. Considering the estimate error of depth-averaged currents and the reality of spatiotemporal ocean currents, additional information and strategies need to be adopted. We focus here on using an acoustic beacon to periodically emit acoustic signals and receiving the emitted signal by a hydrophone installed on the glider. With no need to synchronize the hydrophone time and beacon time, the time difference between two adjacent received signals is utilized to calculate the distance variation from the glider to the beacon, and then the calculated distance variation is set as an additional measurement to estimate the glider location.

## 3. Modified Kinematic Model for the Sea-Wing Underwater Glider

The Sea-Wing underwater glider [61] is driven by an internal buoyancy regulating system and is steered by a movable battery package and a vertical rudder. In this section, we establish a kinematic model for the Sea-Wing underwater glider by considering hydrodynamic parameters to simulate the movement of the glider accurately.

### 3.1. Kinematic Model

**Figure 3:**
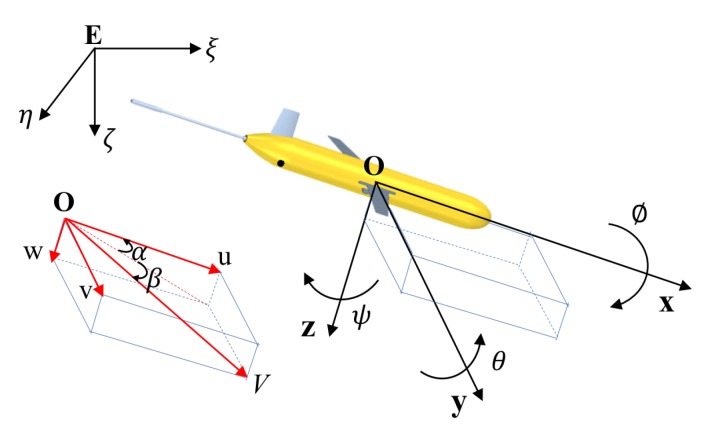
Coordinate frames and motion parameters for the Sea-Wing underwater glider. E:(ξ,η,ζ) represents the inertial frame, and O:(x,y,z) represents the body frame.

To describe the motion of the Sea-Wing underwater glider, two coordinate frames are established based on a right-handed coordinate system, including the inertial frame and body frame (Figure 3). The body frame O:(x,y,z) is established at the buoyancy center of the glider. The x-axis points forward along the longitudinal axis of the glider. The y-axis lies in the wing plane, pointing to the right when viewed along the x direction. The z-axis is set as x×y. The inertial frame E:(ξ,η,ζ) is established at a point in space. A rotation matrix $R_{OE}$ that transforms the body coordinate system into the inertial coordinate system can be defined as

(1) $$R_{OE}=\left[\begin{array}{ccc} c\theta c\psi & s\phi s\theta c\psi -c\phi s\psi & c\phi s\theta c\psi +s\phi s\psi \\ c\theta s\psi & s\phi s\theta s\psi +c\phi c\psi & c\phi s\theta s\psi -s\phi c\psi \\ -s\theta & s\phi c\theta & c\phi c\theta \end{array}\right],$$

in which θ is the pitch angle, ψ is the heading angle and ϕ is the roll angle. The simplified notations c·=cos(·) and s·=sin(·) are used. By the electronic compass installed on the underwater glider, θ, ψ and ϕ can be measured in real time. When the glider glides downward, θ is defined as negative. In addition, ψ is the azimuth relative to the north. When viewed along the x direction, ϕ is negative to the left.

Because there is no velocity sensor, the glider velocity, defined as $V={\left[u,v,w\right]}^{T}$ in the body frame, needs to be estimated from the dynamic model and available measurements. Based on the attack angle α and the drift angle β, the relation among the velocity components can be expressed as

(2) $$\tan\alpha =\frac{w}{u},$$

(3) $$\tan\beta =\frac{v}{u}.$$

The available measurement is the vertical speed of the glider $\dot{\zeta }$ in the inertial coordinate system, which can be calculated through the depth change of the glider measured by the CTD. The relation between the rate of change in position $b={\left[\xi ,\eta ,\zeta \right]}^{T}$ in the inertial frame and the velocity V in the body frame can be written as

(4) $$\dot{b}=R_{OE}V.$$

Substituting Equations (2) and (3) into Equation (4) gives

(5) $$\dot{b}=R_{OE}R_{V}\left|V\right|$$

where

(6) $$R_{V}=\left[\begin{array}{c} c\beta c\alpha \\ s\beta \\ c\beta s\alpha \end{array}\right]$$

and $\left|V\right|=\sqrt{u^{2}+v^{2}+w^{2}}$. From the vertical speed $\dot{\zeta }$, we can derive

(7) $$\left|V\right|={\left(R_{OE}^{\left(3\right)}R_{V}\right)}^{-1}\dot{\zeta }.$$

where $R_{OE}^{\left(i\right)}$ denotes the *i*-th row of $R_{OE}$ and the same denotation is used for the other matrices. Then, the horizontal components of $\dot{b}$ can be calculated from

(8) $${\dot{b}}^{\left(1,2\right)}=R_{OE}^{\left(1,2\right)}R_{V}{\left(R_{OE}^{\left(3\right)}R_{V}\right)}^{-1}\dot{\zeta }.$$

Therefore, α and β are the determining factors for this solution, which can be estimated from the dynamic model of the Sea-Wing underwater glider.

### 3.2. Solution of Attack Angle

**Figure 4:**
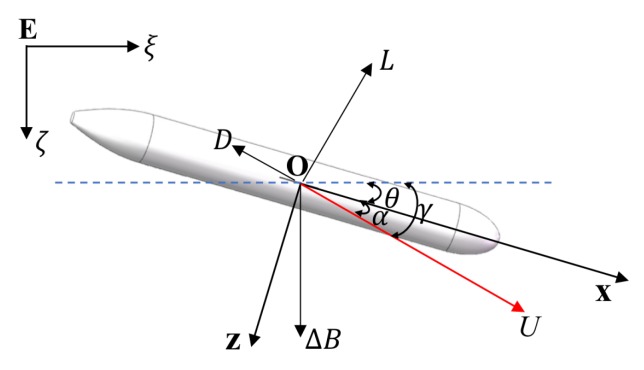
Force analysis model of the Sea-Wing underwater glider in the vertical plane.

The attack angle, α, affects the vertical velocity of the glider. Figure 4 shows the forces of the glider when moving in the vertical plane. Here, γ is the gliding angle, $U=\sqrt{u^{2}+w^{2}}$ is the gliding speed, *L* is the lift force, *D* is the drag force, and ΔB is the residual buoyancy difference.

The steady-state dynamic model of the glider in the vertical plane can be expressed as

(9) ΔBcosγ=−L,

(10) ΔBsinγ=−D,

in which γ=θ+α. In addition, α is defined in the same direction as θ. The hydrodynamic lift and drag forces are modeled in [62] as

(11) $$L=-\left(K_{L0}+K_{L}\alpha \right)U^{2},$$

(12) $$D=-\left(K_{D0}+K_{D}\alpha^{2}\right)U^{2},$$

where $K_{L0}$ and $K_{L}$ are the lift coefficients and $K_{D0}$ and $K_{D}$ are the drag coefficients. Then, we can derive the relation between θ and α as

(13) $$\tan\left(\theta +\alpha \right)=\frac{D}{L}=\frac{K_{D0}+K_{D}\alpha^{2}}{K_{L0}+K_{L}\alpha }.$$

the lift and drag coefficients can be estimated using computational fluid dynamics (CFD) technology. Specifically, for the Sea-Wing underwater glider, $K_{L0}=0.0105$, $K_{L}=496.8596$, $K_{D0}=6.9650$, and $K_{D}=439.7317$.

**Figure 5:**
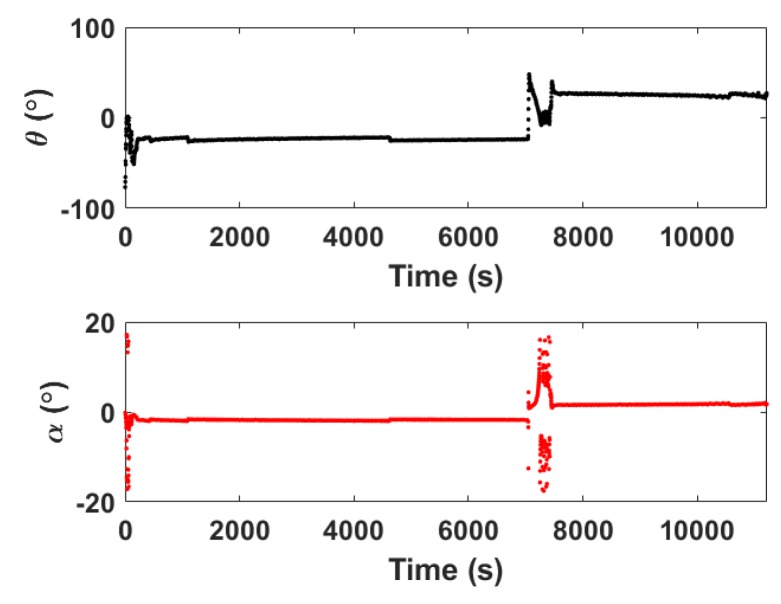
An example of the measured θ and calculated α over the relative time in one gliding cycle during the South China Sea experiment conducted on 13 July 2019.

Because θ can be measured by the electronic compass in real time during the gliding process, α can be calculated based on Equation (13). An example is shown in Figure 5. Since α is not zero, the actual gliding angle is not equal to θ which is usually used for dead reckoning. The sharp change of the angle corresponds to the unsteady state of the glider at the beginning of the gliding cycle and near the inflection point due to state transition. In this case, the measured θ and calculated α are not reliable, so we actually set the angles during the period of unsteady state to interpolation results of adjacent steady-state points.

### 3.3. Solution of Drift Angle

**Figure 6:**
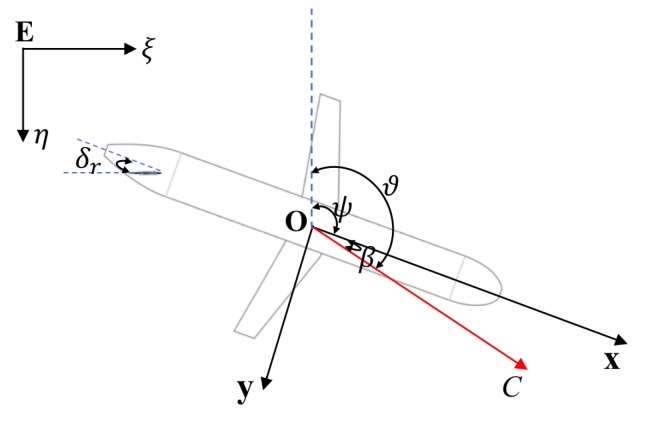
Motion variables of the Sea-Wing underwater glider in the horizontal plane.

The drift angle, β, affects the horizontal velocity of the glider. Figure 6 shows the variables for motion analysis in the horizontal plane. Here, ϑ=ψ+β is the course angle, $C=\sqrt{u^{2}+v^{2}}$ is the horizontal speed, and $\delta_{r}$ is the rudder angle. Rudder rotation changes the direction of the horizontal force, which in turn changes the course of the glider. Based on the analysis for a maneuvering submarine [63], the glider motion with respect to the body coordinate system in the horizontal plane can be represented by

(14) $$\begin{array}{cc} m(\dot{u}-vr) & =\frac{1}{2}\rho l^{4}X_{rr}^{{}^{\prime }}r^{2}+\frac{1}{2}\rho l^{3}\left(X_{\dot{u}}^{{}^{\prime }}\dot{u}+X_{vr}^{{}^{\prime }}vr\right)\\ \; & +\frac{1}{2}\rho l^{2}\left(X_{uu}^{{}^{\prime }}u^{2}+X_{vv}^{{}^{\prime }}v^{2}+X_{\delta_{r}\delta_{r}}^{{}^{\prime }}u^{2}\delta_{r}^{2}\right), \end{array}$$

(15) $$\begin{array}{cc} m(\dot{v}+ur) & =\frac{1}{2}\rho l^{4}(Y_{\dot{r}}^{{}^{\prime }}\dot{r}\\ \; & +Y_{r|r|}^{{}^{\prime }}r|r|)+\frac{1}{2}\rho l^{3}(Y_{\dot{v}}^{{}^{\prime }}\dot{v}+Y_{r}^{{}^{\prime }}ur+Y_{v|r|}^{{}^{\prime }}v|r|)\\ \; & +\frac{1}{2}\rho l^{2}(Y_{0}^{{}^{\prime }}u^{2}+Y_{v}^{{}^{\prime }}uv+Y_{v|v|}^{{}^{\prime }}v|v|+Y_{\delta_{r}}^{{}^{\prime }}u^{2}\delta_{r}), \end{array}$$

(16) $$\begin{array}{cc} I_{zz}\dot{r} & =\frac{1}{2}\rho l^{5}(N_{\dot{r}}^{{}^{\prime }}\dot{r}+N_{r|r|}^{{}^{\prime }}r|r|)\\ \; & +\frac{1}{2}\rho l^{4}(N_{\dot{v}}^{{}^{\prime }}\dot{v}+N_{r}^{{}^{\prime }}ur+N_{v|r|}^{{}^{\prime }}v|r|)\\ \; & +\frac{1}{2}\rho l^{3}(N_{0}^{{}^{\prime }}u^{2}+N_{v}^{{}^{\prime }}uv+N_{v|v|}^{{}^{\prime }}v|v|+N_{\delta_{r}}^{{}^{\prime }}u^{2}\delta_{r}), \end{array}$$

where *m* is the glider mass; ρ is the seawater density; *l* is the glider length; *r* is the angular velocity about the z-axis; $\dot{r}$ is the angular acceleration about the z-axis; $I_{zz}$ is the moment about the z-axis; $\dot{u}$ and $\dot{v}$ are the accelerations in the x and y directions, respectively; and $X_{rr}^{{}^{\prime }},\;Y_{r}^{{}^{\prime }},\;N_{v}^{{}^{\prime }},\cdots$ are nondimensional coefficients of hydrodynamic forces. When the glider is in the steady rotation phase, $\dot{u}=\dot{v}=\dot{r}=0$, and Equations (14)–(16) can be expressed as

(17) $$\begin{array}{cc} 0 & =\frac{1}{2}\rho l^{4}Y_{r|r|}^{{}^{\prime }}r|r|+\frac{1}{2}\rho l^{3}(Y_{r}^{{}^{\prime }}ur+Y_{v|r|}^{{}^{\prime }}v|r|)\\ \; & +\frac{1}{2}\rho l^{2}(Y_{v}^{{}^{\prime }}uv+Y_{v|v|}^{{}^{\prime }}v|v|+Y_{\delta_{r}}^{{}^{\prime }}u^{2}\delta_{r})-mur \end{array}$$

(18) $$\begin{array}{cc} 0 & =\frac{1}{2}\rho l^{5}N_{r|r|}^{{}^{\prime }}r|r|+\frac{1}{2}\rho l^{4}(N_{r}^{{}^{\prime }}ur+N_{v|r|}^{{}^{\prime }}v|r|)\\ \; & +\frac{1}{2}\rho l^{3}(N_{v}^{{}^{\prime }}uv+N_{v|v|}^{{}^{\prime }}v|v|+N_{\delta_{r}}^{{}^{\prime }}u^{2}\delta_{r}) \end{array}$$

According to the assumptions of the linear motion equation, Equations (17) and (18) can be simplified as

(19) $$\left\{\begin{array}{c} \frac{1}{2}\rho l^{2}Y_{v}^{{}^{\prime }}uv-\left(mu-\frac{1}{2}\rho l^{3}Y_{r}^{{}^{\prime }}u\right)r=-\frac{1}{2}\rho l^{2}Y_{\delta_{r}}^{{}^{\prime }}u^{2}\delta_{r}\\ \frac{1}{2}\rho l^{3}N_{v}^{{}^{\prime }}uv+\frac{1}{2}\rho l^{4}N_{r}^{{}^{\prime }}ur=-\frac{1}{2}\rho l^{3}N_{\delta_{r}}^{{}^{\prime }}u^{2}\delta_{r} \end{array}\right.$$

Assuming that β is small, Equation (3) can be approximated as

(20) β=v/u.

Then, the solution of Equation (19) can be derived as

(21) $$\beta =-\frac{N_{\delta_{r}}^{{}^{\prime }}\left(m^{{}^{\prime }}-Y_{r}^{{}^{\prime }}\right)+N_{r}^{{}^{\prime }}Y_{\delta_{r}}^{{}^{\prime }}}{N_{v}^{{}^{\prime }}\left(m^{{}^{\prime }}-Y_{r}^{{}^{\prime }}\right)+N_{r}^{{}^{\prime }}Y_{v}^{{}^{\prime }}}\cdot \delta_{r},$$

where $m^{{}^{\prime }}=m/\left(0.5\rho l^{3}\right)$ is the nondimensional weight. For the Sea-Wing underwater glider, $\delta_{r}\in \left[-30^{\circ }\;30^{\circ }\right]$. Here, $\delta_{r}=0^{\circ }$ represents the x direction, and when viewed along the x direction, $\delta_{r}$ is positive to the left. $\delta_{r}$ is continuously changed during the gliding process to resist the ocean current, to adjust the course angle of the glider, and to keep the preset trajectory as far as possible.

**Figure 7:**
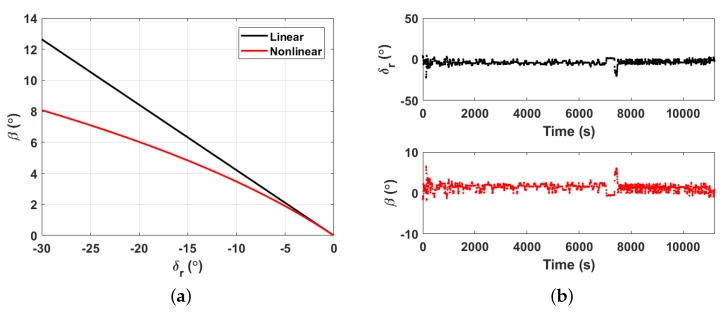
$\delta_{r}$ and β. (**a**) is the relationship and (**b**) is an example of recorded $\delta_{r}$ and calculated β over the relative time in one gliding cycle during the South China Sea experiment conducted on 13 July 2019.

Using CFD software Fluent [64], we can simulate the solutions of β under different $\delta_{r}$. The specific numerical values of the hydrodynamic coefficients used in the simulation are shown in Table 2. Figure 7a shows both the nonlinear results from Equations (17) and (18) and the linear results from Equation (21). As the absolute value of $\delta_{r}$ decreases, the difference between the nonlinear and linear results gradually decreases. Based on real-time adjustment of the glider, $\delta_{r}$ can be acquired to estimate the corresponding value of β, and an example is shown in Figure 7b.

### 3.4. Comparison with Dead Reckoning

For comparison purpose, we compute the dead-reckoning navigation results. The dead reckoning method takes no consideration of α and β, that is α=0 and β=0. Then from Equation (8), the velocity of the underwater glider, ${\dot{b}}_{DR}$, could be calculated by

(22) $${\dot{b}}_{DR}=\left[\begin{array}{c} -\dot{\zeta }\cot\theta c\psi \\ -\dot{\zeta }\cot\theta s\psi \\ \dot{\zeta } \end{array}\right].$$

**Figure 8:**
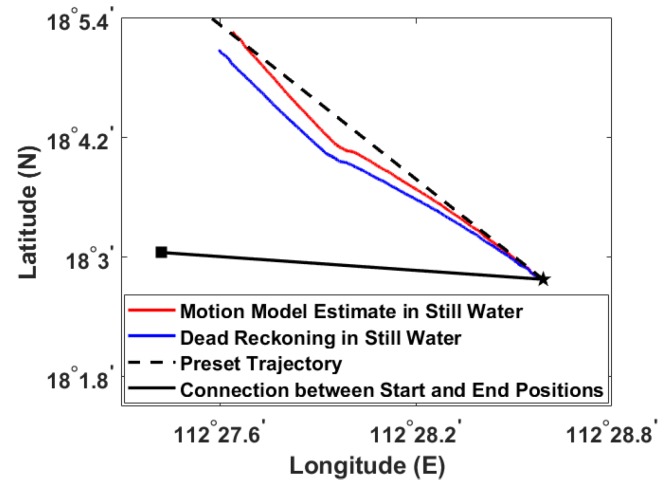
Comparison of navigation results by the proposed kinematic model and dead reckoning based on the data shown in Figure 5 and Figure 7b. The preset trajectory and connection between the actual start and end positions are shown for comparison.

Figure 8 shows an example of dead reckoning result compared with the result of modified kinematic model. The deviation between the preset trajectory and the actual trajectory, represented by the connection between the actual start and end positions, is mainly caused by ocean currents. The estimated trajectory of modified kinematic model is closer to the preset trajectory than the dead reckoning trajectory, indicating that the proposed kinematic model reflects the resistance of the glider to the ocean current through angle adjustment. Then the navigation result of the kinematic model superimposed ocean current can be closer to the real situation.

## 4. EKF-Based Localization and Navigation System Modeling

Based on the established kinematic model in Section 3, we can estimate the moving trajectories of a Sea-Wing underwater glider in still water. With the estimation module of depth-averaged current velocities on the glider, the position of an underwater glider in the ocean can be approximately estimated. The basic approach for estimating the depth-averaged current velocity is to use the difference between the dead-reckoning and GPS positions of resurfacing when a gliding cycle ends, divided by the gliding time [24]. The calculated depth-averaged current velocity is not accurate due to the dead-reckoning navigation error. Even if we adopt a more accurate model, such as the proposed kinematic model, the estimated positions of underwater gliders using depth-averaged current velocities may still be unreliable because of the reality of spatiotemporal ocean currents. Additional acoustic measurements are required to calibrate the estimated positions.

In this section, an EKF method is imported to recursively estimate the positions of the glider during a gliding cycle. The prediction process is based on the modified kinematic model and depth-averaged current velocities, and the update process is based on the distance variation from the glider to the beacon. The RTS smoothing algorithm is adopted to improve the EFK estimation by introducing subsequent measurements.

### 4.1. System State Prediction

Because the glider depth, ζ, can be measured by the installed CTD, the components of glider positions that need to be estimated are ${\left(\xi ,\eta \right)}^{T}$, that is, the east and south components in the horizontal plane. We set the system state vector, s, as below,

$$s={\left(\xi ,\eta \right)}^{T}.$$

Based on Equation (8) and the depth-averaged current velocity, the process model for the system satisfies the following motion equation:

(23) $$\left[\begin{array}{c} \dot{\xi }\\ \dot{\eta } \end{array}\right]=\left[\begin{array}{c} R_{OE}^{\left(1\right)}R_{V}{\left(R_{OE}^{\left(3\right)}R_{V}\right)}^{-1}\dot{\zeta }+v_{x}\\ R_{OE}^{\left(2\right)}R_{V}{\left(R_{OE}^{\left(3\right)}R_{V}\right)}^{-1}\dot{\zeta }+v_{y} \end{array}\right]+\left[\begin{array}{c} g_{1}\\ g_{2} \end{array}\right]w_{s}$$

that is,

(24) $$\dot{s}\left(t\right)=f\left(s\left(t\right),u\left(t\right),v\left(t\right),t\right)+G_{p}w_{p}\left(t\right)$$

where

$$G_{p}=\left[\begin{array}{cc} g_{1} & 0\\ 0 & g_{2} \end{array}\right],\;w_{p}\left(t\right)=\left[\begin{array}{cc} w_{s}\left(t\right) & 0\\ 0 & w_{s}\left(t\right) \end{array}\right],$$

$v\left(t\right)={\left(v_{x},v_{y}\right)}^{T}$ and $u\left(t\right)={\left(\alpha ,\beta ,\theta ,\psi ,\phi ,\delta_{r},\dot{\zeta }\right)}^{T}$. u(t) is the control input for the modified kinematic model. $v_{x}$ and $v_{y}$ are the depth-averaged current components along the ξ and η directions, respectively. $w_{p}\left(t\right)$ is the zero-mean Gaussian process noise, representing accelerations that allow the ocean current to deviate from the depth-averaged current. $G_{p}$ can be considered as a weight matrix, which determines the difference of process noise in different directions.

Then, the propagation of the system state estimate $\hat{s}\left(t\right)$ and error covariance P(t) using the EKF [48] can be written as

(25) $$\dot{\hat{s}}\left(t\right)=f\left(\hat{s}\left(t\right),u\left(t\right),v\left(t\right),t\right)$$

(26) $$\dot{P}\left(t\right)=F\left(t\right)P\left(t\right)+P\left(t\right)F^{T}\left(t\right)+G_{p}QG_{p}^{T},$$

where F(t) is a system dynamic matrix,

(27) $$F\left(t\right)\equiv \frac{\partial f}{\partial s}{|}_{\hat{s}\left(t\right)}=\left[\begin{array}{cc} 0 & 0\\ 0 & 0 \end{array}\right],$$

and the process-noise matrix Q can be written as

(28) $$Q=\left[\begin{array}{cc} \Phi_{s} & 0\\ 0 & \Phi_{s} \end{array}\right]$$

under the consideration that each direction of the ocean current accelerations is uncorrelated. $\Phi_{s}$ is the spectral density of the white noise $w_{s}$.

### 4.2. Measurement Model

A fixed beacon with a known position is needed to transmit signals at regular intervals. The Sea-Wing underwater glider equipped with hydrophones can receive the acoustic signal emitted from the preset acoustic beacon. Assuming that the emitted acoustic signal propagates along a straight line, the distance between the receiver and the beacon can be calculated by the product of the travel-time measurement and the sound speed in water. Time synchronization between the receiver and the beacon is necessary in order to obtain an accurate travel time. To overcome this constraint, we propose here to utilize the time difference between two adjacent receiving locations. Specifically, as long as the beacon transmits a signal according to a preset time interval Δt, the receiver can calculate the travel-time difference according to the receiving time of two adjacent signals, thereby calculating the distance difference.

The range from the beacon to the receiver is a nonlinear function of the receiver location and the beacon location. At a time point, $t_{k}\;\left(k=1,2,\ldots \right)$, the location of the receiver, that is, the glider, is $s_{k}={\left(\xi_{k},\eta_{k}\right)}^{T}$, and the beacon location is $s_{bk}={\left(\xi_{bk},\eta_{bk}\right)}^{T}$. The perfect range measurement can be written as

(29) $$R_{k}=\sqrt{{\left(s_{k}-s_{bk}\right)}^{T}\left(s_{k}-s_{bk}\right)}.$$

The distance difference corresponding to two adjacent receiving times, $t_{k}$ and $t_{k-j}\;\left(k\geq K+1,j=1,\ldots ,K\right)$, can be modeled as

(30) $$\Delta R_{k}^{\left(j\right)}=R_{k}-R_{k-j}.$$

Then considering the actual noisy range difference measurement, ${\tilde{y}}_{k}$, we can set the measurement model as

(31) $${\tilde{y}}_{k}=h\left(s_{k}\right)+n_{k}$$

where

(32) $${\tilde{y}}_{k}^{\left(j\right)}=\Delta R_{k}^{\left(j\right)}+n_{k}^{\left(j\right)}=c\left(t_{k}-t_{k-j}-j\Delta t\right),$$

c=1500 m/s, and *K* is the virtual array size set by experience. $n_{k}∼N\left(0,N_{r}\right)$ is the measurement noise, which is in units of distance and represents the imprecision in timing multiplied by *c*. The variance $N_{r}$ is assumed to be independent of time. The Jacobian of the range difference measurement, Equation (31), at $t_{k}$ with respect to s is $H_{k}\equiv \frac{\partial h}{\partial s}{|}_{{\hat{s}}_{k}}$ with

(33) $$H_{k}^{\left(j\right)}={\left[\begin{array}{c} \frac{\xi_{k}-\xi_{bk}}{R_{k}}-\frac{\xi_{k-j}-\xi_{b(k-j)}}{R_{k-j}}\\ \frac{\eta_{k}-\eta_{bk}}{R_{k}}-\frac{\eta_{k-j}-\eta_{b(k-j)}}{R_{k-j}} \end{array}\right]}^{T}.$$

### 4.3. Recursive Estimation of the System State

EKF methods can operate recursively on noisy input data to produce a statistically suboptimal estimate of the system state [65]. When the range difference measurements are available, the system states are updated. If no measurement is acquired, the system states are predicted by the propagation model, Equation (25), which performs over the sampling step $T_{s}$.

Given the state vector at time point $t_{i-1}=\left(i-1\right)T_{s},\;\left(i=2,3,\ldots \right)$, *a priori* state estimation ${\hat{s}}_{i}^{-}$ by integrating Equation (25) is performed. If at $t_{i}$, the *k*-th (k=K+1,K+2,…) beacon emitted signal is received, then the range-difference measurements ${\tilde{y}}_{k}$ are available. From the *a priori* state estimation ${\hat{s}}_{i}^{-}$ and the estimated state at previous time point $t_{k-j}$, we model the range-difference measurements by Equations (29)–(31). By weighting the deviation between the model-calculated range differences $h({\hat{s}}_{i}^{-})$ and the true range-difference measurements ${\tilde{y}}_{k}$, we can correct the *a priori* state estimate ${\hat{s}}_{i}^{-}$ to acquire an *a posteriori* state estimate ${\hat{s}}_{i}^{+}$ using

(34) $${\hat{s}}_{i}^{+}={\hat{s}}_{i}^{-}+K_{k}\left[{\tilde{y}}_{k}-h\left({\hat{s}}_{i}^{-}\right)\right],$$

where

(35) $$K_{k}=P_{i}^{-}H_{k}^{T}\left({\hat{s}}_{i}^{-}\right){\left[H_{k}\left({\hat{s}}_{i}^{-}\right)P_{i}^{-}H_{k}^{T}\left({\hat{s}}_{i}^{-}\right)+N_{r}\right]}^{-1}$$

is the Kalman gain corresponding to the *k*-th measurement and $P_{i}^{-}$ is *a priori* estimate of the error covariance matrix calculated from Equation (26). The Kalman gain $K_{k}$ is regarded as a weighting function to make the EKF algorithm recursive. Predicted system states with smaller uncertainty, that is, ${\hat{s}}_{i}^{-}$ with smaller $P_{i}^{-}$, are given more weight. The weighted result ${\hat{s}}_{i}^{+}$ and its covariance, which is calculated by inform the prediction used in the following time step.

(36) $$P_{i}^{+}=\left[I-K_{k}H_{k}\left({\hat{s}}_{i}^{-}\right)\right]P_{i}^{-},$$

This online recursive estimation process for a diving cycle of underwater gliders starts from the initial position at the sea surface and continues until the glider rises to the surface again. The purpose of this process is to make the glider know its position and then to better detect the target [6]. The limitation of this EKF system is that the state estimation error introduced by the inaccurate depth-averaged current velocity, which is estimated from the previous gliding cycle, cannot be fully corrected and the cumulative error will increase over time.

### 4.4. Estimation Improvement by RTS Smoothing

To reduce the EKF estimation error, we introduce the RTS smoothing method to the EKF system. Measurements acquired at all available future and past time points are used to estimate the system state. Because additional measurements are utilized in the estimation algorithm, the RTS smoothing intuitively gives better estimates [49,66] to support the spatiotemporal field construction of target radiation signal [7]. Smoothing is the postprocessing process. We can calculate the depth-averaged current velocity for the current gliding cycle from the GPS-localized resurfacing position of the glider, which further reduces the state estimation error.

Let ${\hat{s}}_{s}$ denote the smoothed estimation. Based on the EKF estimated results ${\hat{s}}_{i}^{+}$, the RTS smoothed result ${\hat{s}}_{si}^{+}$ can be estimated by the dynamic model, $F_{i}$, with a correction term, $K_{si}$, as below,

(37) $${\hat{s}}_{si}^{+}={\hat{s}}_{si}^{-}-\left(F_{i}+K_{si}\right)\left({\hat{s}}_{si}^{-}-{\hat{s}}_{i}^{+}\right),$$

where

(38) $${\hat{s}}_{si}^{-}={\hat{s}}_{s(i+1)}^{+}-f\left({\hat{s}}_{s}\left(t\right),u\left(t\right),v\left(t\right),t\right){|}_{t_{i+1}}\left(t_{i+1}-t_{i}\right),$$

and

(39) $$K_{si}\equiv G_{p}QG_{p}^{T}{\left(P_{i}\right)}^{-1}$$

is the smoothed gain. When ${\tilde{y}}_{k}$ is available, $P_{i}=P_{i}^{+}$; otherwise, $P_{i}=P_{i}^{-}$.

Although for the RTS smoothing process, we use the depth-averaged current velocity in the current gliding cycle, there is still a gap with the spatiotemporal flow field that actually affects the position of the glider. To alleviate the problems caused by accumulation errors, we divide a gliding cycle into two parts by taking the inflection point as the boundary. For the second half of the gliding cycle, that is, after the inflection point, the RTS smoothing method is used to perform the estimation on the EKF results from the GPS-localized resurfacing position. For the first half, we still use the EKF results from the initial GPS-localized position with the depth-averaged current velocity in the current gliding cycle. Therefore, it is inevitable that a larger error within the gliding cycle appears near the inflection point. We call this combined estimate the RTS-EKF Estimate.

## 5. Simulation Based on Experimental and Model Data

**Figure 9:**
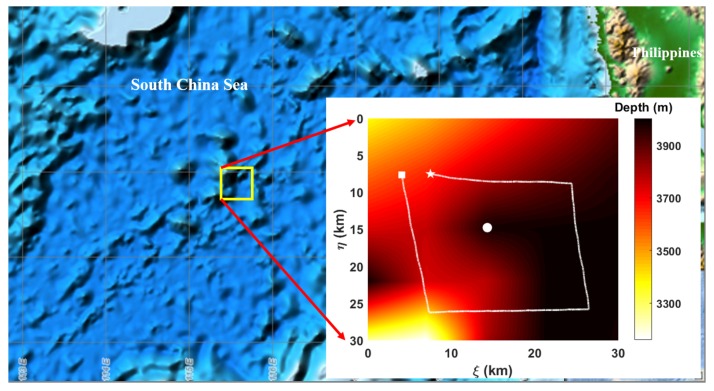
Simulation site. The beacon position (marked by the white dot) and glider trajectory (represented by the white line) are superimposed on the bathymetry data within the yellow box. The white pentagram and square are the starting and ending points for glider operations, respectively.

Simulations show the experimental scene of a Sea-Wing underwater glider in a sea region with a submersible mooring beacon. The simulation site is shown in Figure 9. The number of gliding cycles for the entire operation is 16. The real trajectories of the underwater glider were generated by our proposed kinematic model and simulated ocean currents. The angle and depth data for the kinematic model in the first gliding cycle were based on records recorded during an experiment conducted in the South China Sea because we had no measurement model for the electronic compass and CTD sensor installed on the underwater glider, while the data used in subsequent gliding cycles were based on the conversion of the adopted data in the first cycle. Ocean currents in the South China Sea experimental site had no influence on the accuracy of these adopted angle and depth data. The acoustic data for the EKF-based localization and navigation algorithm were generated by an acoustic simulator.

### 5.1. Simulated Ocean Currents

**Figure 10:**
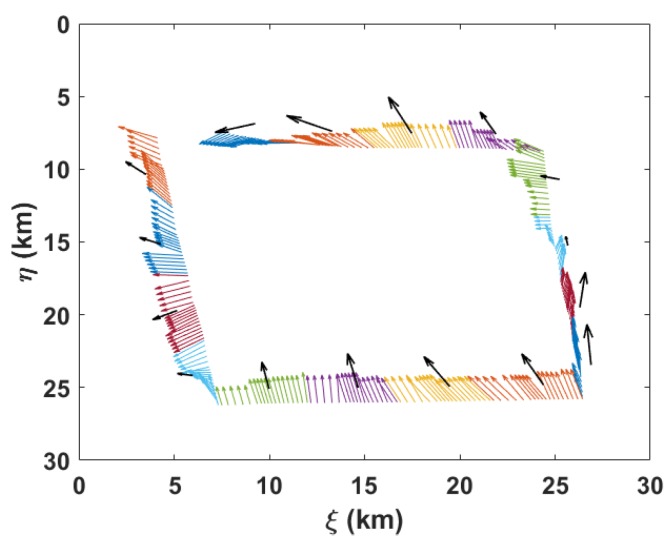
Flow along the glider trajectory. Colored arrows represent real flows for different gliding cycles. Each black arrow is the calculated depth-averaged current for each gliding cycle.

To simulate the motion of an underwater glider in a flow field, we generated a 3D spatiotemporal flow field based on the hydrological basic data acquired from an ocean model, named POM [67]. The generated flow field was combined with the modified kinematic model of the glider for real position setting. Figure 10 shows the flow along the glider trajectory, which reflects that the simulated spatiotemporal flow field varies drastically during the glider operation.

### 5.2. Acoustic Travel-Time Simulation

A virtual acoustic beacon was deployed within the operation region. To simulate the transmission of acoustic signals emitted by the acoustic beacon, we set the environmental parameters. The bathymetry data in the region are obtained from geospatial data at the NOAA website [68]. The sound speed distribution in this region is calculated from the basic hydrological data acquired from a POM South China Sea $1/15^{\circ }$ analysis provided by the South China Sea Institute of Oceanology, Guangzhou, P.R. China [67]. The acoustic travel time of the emitted signals is generated using the toolbox Bellhop 3D, which is published by the Ocean Acoustics Library [69].

The transmission time interval of the acoustic beacon, Δt, is set to 10 s. However, considering the simulation situation, we need to obtain the acoustic travel time based on the glider position. Therefore, we set the receiving interval for the underwater glider to 10 s instead of the beacon emitting interval. The travel time of the direct-path wave can be acquired for each receiving location. Then, the travel-time differences between adjacent receiving locations can be calculated, which are taken as the system measurements by multiplying the sound velocity. So instead of calculating the noisy range-difference measurement ${\tilde{y}}_{k}$ by Equation (32), in simulations, ${\tilde{y}}_{k}$ is acquired by

(40) $${\tilde{y}}_{k}^{\left(j\right)}=c\left(\tau_{k}-\tau_{k-j}\right),$$

where $\tau_{k}\;\left(k\geq K+1,\;j=1,\ldots ,K\right)$ is the simulated one-way travel time corresponding to the receiving time $t_{k}$.

### 5.3. EKF Estimation

The EKF needs to be initialized on startup. The initial value of the system state ${\hat{s}}_{0}$ is set to the real GPS location. The depth-averaged current velocity for the first gliding cycle is set to $v_{x}{{|}_{0}=v_{y}|}_{0}=0$. For the *m*-th (m=2,3,…) gliding cycle, the adopted depth-averaged current velocity is calculated from the (m−1)-th gliding cycle, that is, $(v_{x}{|}_{m-1},v_{y}{{|}_{m-1})}^{T}$. Considering the actual situation, we introduce zero-mean Gaussian noise $\mu ∼N(0,N_{v})$ into the real depth-averaged current velocity. Here, $N_{v}={\left(0.05\;m/s\right)}^{2}$.

The combined effect of $G_{p}$ and Q explains the error induced by the depth-averaged current velocity. We set Q to be a constant matrix with $\Phi_{s}=2\times {\left(0.2\;m/s\right)}^{2}$ for all gliding cycles. $G_{p}$, regarded as a weighting matrix, is assigned varying values for different gliding cycles based on the acceleration of the depth-averaged current velocity. For the first cycle, without any prior knowledge of the current, we set $g_{1}=g_{2}=0.5$. For the *m*-th (m=2,3,…) gliding cycle, the weights $g_{1}$ and $g_{2}$ are set to

(41) $$g_{1}=\frac{a_{x}{|}_{m-1}}{{a|}_{m-1}},\;\mathrm{and}\;g_{2}=\frac{a_{y}{|}_{m-1}}{{a|}_{m-1}},$$

where

(42) $$a_{x}{|}_{m-1}=\frac{\left|v_{x}{{|}_{m-1}-v_{x}|}_{m-2}\right|}{T_{m-1}},$$

(43) $$a_{y}{|}_{m-1}=\frac{\left|v_{y}{{|}_{m-1}-v_{y}|}_{m-2}\right|}{T_{m-1}},$$

(44) $${a|}_{m-1}=a_{x}{{|}_{m-1}+a_{y}|}_{m-1},$$

and $T_{m-1}$ is the gliding period for the (m−1)-th gliding cycle.

The measurement noise variance $N_{r}$ also needs to be given. We set $N_{r}={\left(50\;m\right)}^{2}$ to account for the uncertainty during ranging based on the time difference multiplied by *c*.

**Figure 11:**
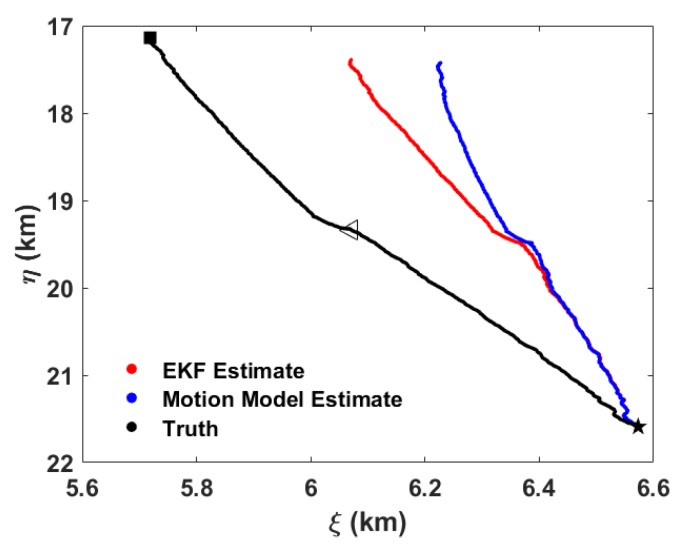
Horizontal positions of the underwater glider in the 14-th gliding cycle. Red dots are the locations estimated by the extended Kalman filter (EKF) method, while blue dots are the results of the motion model. The black dots are real positions of the glider, in which the black pentagram represents the starting point, the black square represents the ending point and the black triangle represents the inflection point.

Because the calculation of depth-averaged current velocity does not rely on the EKF process, we use the modified kinematic model with the superimposed depth-averaged current velocity for comparison, labeled as “Motion Model Estimate” in Figure 11. The trajectory estimated by the EKF method is closer to the real trajectory than the motion model estimate. At the resurfacing position, the estimated error is reduced from 800 m to 630 m. However the estimated trajectory is still far from the actual trajectory.

### 5.4. RTS-EKF Estimation

The RTS backward smoothing algorithm is applied to improve the EKF estimate. For postprocessing, the resurfacing position of the glider is known. Then, the depth-averaged current velocity for the current gliding cycle can be calculated. Therefore, for the *m*-th (m=1,2,…) gliding cycle, the adopted depth-averaged current velocity is $(v_{x}{|}_{m},v_{y}{{|}_{m})}^{T}$. The weights $g_{1}$ and $g_{2}$ are assigned to

(45) $$g_{1}=\frac{a_{x}{|}_{m}}{{a|}_{m}},\;\mathrm{and}\;g_{2}=\frac{a_{y}{|}_{m}}{{a|}_{m}},$$

where

(46) $$a_{x}{|}_{m}=\frac{\left|v_{x}{{|}_{m}-v_{x}|}_{m-1}\right|}{T_{m}},$$

(47) $$a_{y}{|}_{m}=\frac{\left|v_{y}{{|}_{m}-v_{y}|}_{m-1}\right|}{T_{m}},$$

(48) $${a|}_{m}=a_{x}{{|}_{m}+a_{y}|}_{m},$$

and $T_{m}$ is the gliding period for the *m*-th gliding cycle.

**Figure 12:**
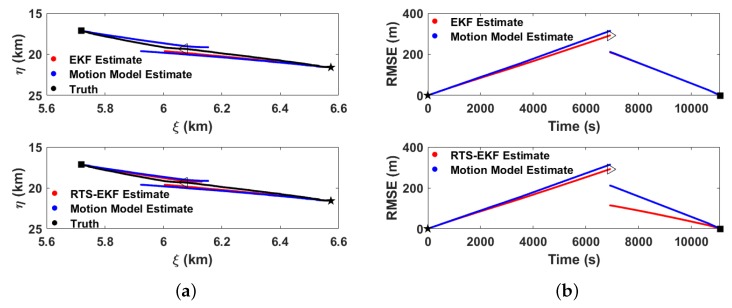
Estimate Results. (**a**) Estimate state and (**b**) Root-mean-square error (RMSE) results. Upper panel: EKF estimate compared with the motion model estimate and the actual condition. Lower panel: Rauch-Tung-Striebel (RTS)-EKF estimate compared with motion model estimate and the actual condition.

Considering the accumulation of errors, we use the RTS smoothing from Equations (37)–(39) for the second half of the gliding cycle, that is, after the inflection point, and for the first half of the gliding cycle, we keep the EKF estimate with $(v_{x}{|}_{m},v_{y}{{|}_{m})}^{T}$. The performance of the estimate state $\hat{s}$ is evaluated by the root-mean-square error (RMSE) as follows:

(49) $$RMSE=\sqrt{\frac{1}{N}{\left(\hat{s}-s\right)}^{T}\left(\hat{s}-s\right)},$$

where *N* is the number of trials. Figure 12 shows the estimate results with N=1. For comparison, the motion model estimate and the EKF estimate for the second half of the gliding cycle is calculated by integrating the dynamic model in reverse time from the resurfacing position.

Because we divide the gliding cycle into two parts, a larger error within the gliding cycle appears near the inflection point. At the inflection point, the RMSE of the RTS-EKF estimate is reduced from 211.4 m to 114.1 m compared with the EKF result, and when compared with the motion model estimate, the performance of the RTS-EKF estimate is improved by 46%. The reason for RTS performance improvement is that the cumulative estimation error of the EKF from the starting point is corrected, and new measurements up to the resurfacing position are introduced.

### 5.5. Discussion

The main error induced to the model-aided estimation process is due to the depth-averaged current velocity. Therefore, except the spatiotemporal characteristics of the ocean current, the error μ of the estimated depth-averaged current velocity impacts the estimate performance. We discussed one trial for the case of $N_{v}={\left(0.05\;m/s\right)}^{2}$ in Section 5.4. Here, multiple trials are conducted for the case of $N_{v}={\left(0.05\;m/s\right)}^{2}$ and for other cases as $N_{v}=\left(0,0.1,0.15,0.2\right)\;{\left(m/s\right)}^{2}$ to explore the statistical performance of the RTS-EKF estimate.

**Figure 13:**
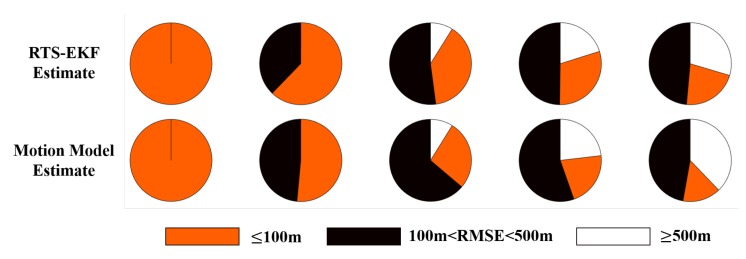
Estimated RMSE over different error values μ of the depth-averaged current velocity. From left to right, the variance $N_{v}=\left(0,0.05,0.1,0.15,0.2\right)\;{\left(m/s\right)}^{2}$. The first row shows the RTS-EKF estimate, and the second row shows the motion model estimate for comparison.

Figure 13 shows the RMSE results over different $N_{v}$. The specific percentages are given in Table 3. For each $N_{v}$, we calculate the percentages of the RMSE values within different error ranges for all gliding cycles. The motion model estimate is shown for comparison. As $N_{v}$ increases, the percentage for RMSE≤ 100 m gradually decreases, while the percentage for RMSE≥ 500 m gradually increases. Under the same case of $N_{v}$, the percentage for RMSE≤ 100 m corresponding to the RTS-EKF estimate is always larger than that of the motion model estimate, while the percentage for RMSE≥ 500 m corresponding to the RTS-EKF estimate is always not greater than that of the motion model estimate.

## 6. Conclusions

This paper has formulated a dynamic localization and navigation method for underwater gliders based on a modified kinematic model combined with acoustic measurements from a single beacon. The adopted acoustic measurement is based on the travel-time differences between adjacent signals from the beacon, multiplied by the sound speed in water. Therefore, there is no need to synchronize the hydrophone time and beacon time. The depth-averaged current velocity is introduced into the modified kinematic model to generate a state estimate of underwater gliders in a flow field. An EKF method is preformed based on the estimated state and the calculated range differences. To improve the EKF estimate, the RTS smoothing algorithm is adopted for each gliding cycle after the inflection point by introducing subsequent measurements.

From simulation results, the EKF method can estimate the glider positions better than the motion model. However, the estimated glider resurfacing point is still 630 m from the actual resurfacing position. The proposed RTS-EKF method can further reduce the gap between the estimated trajectory and the true trajectory from 211.4 m to 114.1 m at the inflection point compared with the EKF result. When compared with the motion model estimate, the performance of the RTS-EKF estimate is improved by 46% at the inflection point. The influence of the error of the adopted depth-averaged current velocity is discussed, which further illustrates the superior performance of the proposed RTS-EKF method.

## Figures and Tables

**Table 1:** Some Specifications of the Sea-Wing Underwater Glider.

Size (m)	Hull diameter 0.22, vehicle length 2, wing span 1.2
Weight (kg)	65
Inflection Depth (m)	1000
Cruising Speed (m/s)	0.25, maximum 0.5
Range (km)	>1100
Communications	Iridium and radio communication
Navigation	Global positioning system, altimeter and electronic compass
Science Sensor	CTD and hydrophone

**Table 2:** Nondimensional coefficients of hydrodynamic forces used for simulations.

Coefficients	$Y_{r|r|}^{{}^{\prime }}$	$Y_{r}^{{}^{\prime }}$	$Y_{v|r|}^{{}^{\prime }}$	$Y_{v}^{{}^{\prime }}$	$Y_{v|v|}^{{}^{\prime }}$	$Y_{\delta_{r}}^{{}^{\prime }}$
Values	0.00611	0.01065	−0.03931	−0.03545	0.01558	−0.00968
Coefficients	$N_{r|r|}^{{}^{\prime }}$	$N_{r}^{{}^{\prime }}$	$N_{v|r|}^{{}^{\prime }}$	$N_{v}^{{}^{\prime }}$	$N_{v|v|}^{{}^{\prime }}$	$N_{\delta_{r}}^{{}^{\prime }}$
Values	−0.00303	−0.00523	0.01312	−0.00149	−0.01983	0.00368

**Table 3:** RMSE comparison over different errors of the depth-averaged current velocity.

$\sqrt{N_{v}}$ m/s	RTS-EKF Estimate			Motion Model Estimate		
	≤100 m	100 m<·<500 m	≥500 m	≤100 m	100 m<·<500 m	≥500 m
0	100%	0	0	100%	0	0
0.05	62%	38%	<1%	51%	49%	<1%
0.1	39%	52%	9%	27%	64%	9%
0.15	30%	50%	20%	21%	55%	24%
0.2	22%	49%	30%	15%	47%	38%
